# Experimental rhinovirus infection induces an antiviral response in circulating B cells which is dysregulated in patients with asthma

**DOI:** 10.1111/all.14985

**Published:** 2021-07-16

**Authors:** Oliver F. Wirz, Kirstin Jansen, Pattraporn Satitsuksanoa, Willem van de Veen, Ge Tan, Milena Sokolowska, David Mirer, Barbara Stanić, Simon D. Message, Tatiana Kebadze, Nicholas Glanville, Patrick Mallia, James E. Gern, Nikolaos Papadopoulos, Cezmi A. Akdis, Sebastian L. Johnston, Kari Nadeau, Mübeccel Akdis

**Affiliations:** ^1^ Swiss Institute of Allergy and Asthma Research (SIAF) University of Zurich Davos Switzerland; ^2^ Christine Kühne – Center for Allergy Research and Education (CK‐CARE) Davos Switzerland; ^3^ Functional Genomics Center Zürich ETH Zürich/University of Zürich Zürich Switzerland; ^4^ National Heart and Lung Institute Imperial College London London UK; ^5^ Department of Pediatrics University of Wisconsin‐Madison Madison USA; ^6^ Division of Infection, Immunity & Respiratory Medicine The University of Manchester Manchester UK; ^7^ Allergy Department 2nd Pediatric Clinic University of Athens Athens Greece; ^8^ Sean N. Parker Center for Allergy and Asthma Research Department of Medicine Stanford University Palo Alto California USA; ^9^ Present address: Department of Pathology Stanford University Stanford California USA

**Keywords:** allergic asthma, B lymphocytes, interferon‐stimulated genes (ISG), pro‐inflammatory cytokines, rhinovirus infection

## Abstract

**Background:**

Rhinoviruses are the predominant cause of respiratory viral infections and are strongly associated with asthma exacerbations. While humoral immunity plays an important role during virus infections, cellular aspects of this response are less well understood. Here, we investigated the antiviral response of circulating B cells upon experimental rhinovirus infection in healthy individuals and asthma patients.

**Methods:**

We purified B cells from experimentally infected healthy individuals and patients with asthma and subjected them to total RNA‐sequencing. Rhinovirus‐derived RNA was measured in isolated B cells using a highly sensitive PCR. B cells were stimulated with rhinovirus *in vitro* to further study gene expression, expression of antiviral proteins and B‐cell differentiation in response rhinovirus stimulation. Protein expression of pro‐inflammatory cytokines in response to rhinovirus was assessed using a proximity extension assay.

**Results:**

B cells isolated from experimentally infected subjects exhibited an antiviral gene profile linked to IFN‐alpha, carried viral RNA *in vivo* and were transiently infected by rhinovirus *in vitro*. B cells rapidly differentiated into plasmablasts upon rhinovirus stimulation. While B cells lacked expression of interferons in response to rhinovirus exposure, co‐stimulation with rhinovirus and IFN‐alpha upregulated pro‐inflammatory cytokine expression suggesting a potential new function of B cells during virus infections. Asthma patients showed extensive upregulation and dysregulation of antiviral gene expression.

**Conclusion:**

These findings add to the understanding of systemic effects of rhinovirus infections on B‐cell responses in the periphery, show potential dysregulation in patients with asthma and might also have implications during infection with other respiratory viruses.

## Graphical abstract

Intranasal infection with rhinovirus‐A16 elicits antiviral response in peripheral B cells, characterized by upregulated antiviral response and cytokine genes. Peripheral B cells are dependent on external type‐I‐IFN to develop an antiviral activation state. Rhinovirus stimulation induces strong antiviral response in plasmablasts. Dysregulated expression of antiviral response and antibody genes in individuals with asthma is observed after rhinovirus infection. Abbreviations: IFI44L, interferon induced protein 44 like; IFIT, interferon induced protein with tetratricopeptide repeats; IGHA, immunoglobulin heavy constant alpha; IGHG, immunoglobulin heavy constant gamma; IFN‐ α, IFN‐alpha; MX, MX dynamin like GTPase; OASL, 2'‐5'‐oligoadenylate synthetase like; RV‐A16, rhinovirus‐A16; STAT1, signal transducer and activator of transcription 1

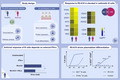

**Abbreviations**
APC: Antigen‐presenting cells
BAL: Bronchoalveolar lavage
BCR: B‐cell receptor
bp: base pairs
CD: Cluster of differentiation
*DDX58*
: Gene encoding Retinoic acid‐inducible gene I
FEV_1_
: Forced expiratory volume in 1 s
FVC: Forced vital capacity
*HERC5*
: Gene encoding HECT And RLD Domain Containing E3 Ubiquitin Protein Ligase 5
HRV: Human rhinovirus
ICAM‐1: Intercellular adhesion molecule 1
*IFI44L*
: Gene encoding interferon‐induced protein 44 Like
*IFIT1*
: Gene encoding interferon‐induced protein with tetratricopeptide repeats 1
*IFITM1*
: Gene encoding interferon‐induced transmembrane protein 1
IFN: Interferon
Ig: Immunoglobulin
*MX1*
: Gene encoding interferon‐induced GTP‐binding protein Mx1
NGS: Next generation RNA‐sequencing
PBMC: Peripheral blood mononuclear cells
PCR: Polymerase chain‐reaction
PEF: Peak expiratory flow
RNA: Ribonucleic acid
RT‐PCR: Real‐time polymerase chain‐reaction
RV: Rhinovirus
RV‐A16: Rhinovirus strain 16
RV‐A39: Rhinovirus strain 39
ssRNA: single‐stranded RNA
*STAT1*
: Gene encoding signal transducer and activator of transcription 1
TLR: Toll‐like receptor
UTR: Untranslated region

## 1 INTRODUCTION

Around 50%–70% of upper respiratory tract infections are caused by human rhinoviruses (RV), making them the most common cause of viral‐induced respiratory diseases and a major healthcare burden.^1^, ^2^ RV infections are usually not life‐threatening for healthy individuals, and up to 30% of infections are asymptomatic.^3^, ^4^, ^5^ Rhinoviruses replicate primarily in epithelial cells of the upper and lower respiratory tract.^6^, ^7^, ^8^ Together with phagocytes from mucosa‐associated lymphoid tissues, these cells produce an array of pro‐inflammatory cytokines, including type‐I interferons (IFN). While type‐I IFNs are essential mediators to induce antiviral immunity, they also play an important role in various B‐cell differentiation processes.^9^, ^10^, ^11^, ^12^, ^13^

B cells are constantly circulating through the body's tissues and are also present at the mucosal sites of the respiratory tract, where RV infections mainly take place.^14^, ^15^ B cells also represent the major cell population in tonsils^16^ where RV can be detected.^17^ B cell–mediated humoral immune response plays a central role in controlling RV infections^18^, ^19^ with neutralizing serum IgG and secretory IgA in the mucosa detected one to 2 weeks after infection, with a role of protection from re‐infection with the same strand.^18^, ^19^ B cells were also found to internalize RV particles and proliferate in response to RV stimulation *in vitro*.^20^ B cells crucially contribute to eliciting adaptive immune responses by transporting lung‐derived viral and bacterial antigens to secondary lymphoid organs in various murine models.^21^, ^22^, ^23^

While RV infections usually lead to mild symptoms in healthy adults, they can lead to complications in young children or in adults with underlying chronic respiratory diseases, particularly asthma. RV‐induced wheezing in early life is strongly associated with the development of asthma during later childhood.^24^ Furthermore, RV infections are the main cause of asthma exacerbations.^25^ Moreover, a less efficient B‐cell response was found in patients with asthma due to a bias towards non–virus‐neutralizing antibodies.^26^, ^27^

Given the frequent incidence of RV infections and the central role of B cells during antiviral responses, it is surprising that the cellular side of this response was not yet addressed in more detail. Therefore, this study aimed to identify the underlying gene regulatory networks driving the B‐cell response during RV infection *in vivo*; to define the external stimulating factors driving this response; to address whether B cells directly interact with RV *in vivo*; and to discover potential B‐cell functions in addition to well‐studied antibody production. Furthermore, since responses to RV were described as less efficient in patients with chronic respiratory diseases, we addressed possible dysregulation of circulating B cells in asthma patients. Here, we report early upregulation of an antiviral gene programme, followed by subsequent upregulation of a pro‐inflammatory response in B cells from experimentally infected human individuals. B cells carried viral RNA after RV infection *in vivo*, suggesting direct interactions of B cells with infecting virions. An elevated antiviral response and broad upregulation of antibody genes in B cells were found in asthma patients.

## 2 METHODS

A detailed description of the study design, sample preparation, methods used for cell culture, flow cytometric cell sorting, quantitative PCR, detection of RV in human samples, measurement of proteins and methods for RNA‐sequencing and data analysis can be found in Appendix S1 section.

In brief, B cells were purified from healthy human subjects that were experimentally infected with RV‐A16. Gene expression of sorted B cells was analysed using RNA‐sequencing. To confirm results from this first experiment, RNA‐sequencing and quantitative PCR were also performed on B cells cultured with RV and IFN‐α *in vitro*. RV‐induced protein expression of antiviral genes and B‐cell subsets were analysed using flow cytometry. Expression of pro‐inflammatory cytokines was measured using a proximity extension assay targeting 92 inflammatory mediators. Gene expression from B cells sorted from experimentally infected asthma patients was compared to gene expression in healthy individuals.

## 3 RESULTS

### 3.1 Intranasal infection with Rhinovirus‐A16 elicits antiviral response in peripheral B cells

**FIGURE 1:**
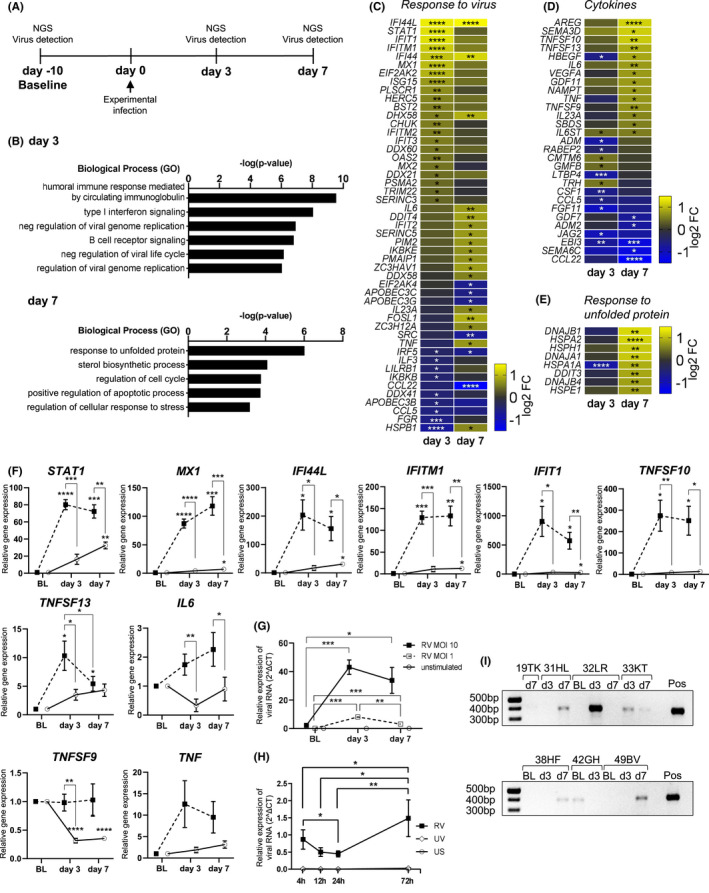
Intranasal infection with rhinovirus‐A16 elicits antiviral response in peripheral B cells of healthy individuals. A, Experimental layout of experimental RV infection experiment. Gene expression of B cells sorted from experimentally RV‐infected non‐asthmatic human subjects was analysed using RNA‐sequencing (B–E). B, Significantly upregulated genes compared to baseline (*n* = 4) on day 3 (*n* = 6) and day 7 (*n* = 6) were analysed for pathway enrichment according to GO Biological Process. C, D, E, Gene expression change shown for virus response genes (C), cytokine genes (D) and response to unfolded protein genes (E). F, G, H, B cells were sorted from *in vitro* RV‐infected PBMC of healthy individuals, and antiviral gene expression (F) or viral RNA expression (G) was analysed using qPCR, *n* = 7. H, *In vitro* cultured PBMC were washed 4h after RV infection to remove excess RV virions from cell culture, B cells were sorted at indicated time points and viral RNA expression analysed using qPCR, *n* = 6. I, RV RNA was detected using a highly sensitive two‐step pan‐RV PCR. Values are means ± SEM, for *in vitro* experiments (F–H), RV was used at multiplicity of infection (MOI) of 10 (10 infectious particles per cell), unless noted otherwise

Immune activation in total peripheral blood cells was observed after infection with respiratory viruses, including influenza, respiratory syncytial virus and rhinovirus (RV).^28^ By producing virus‐neutralizing antibodies, B cells play a central role in the immune response to RV infections.^15^, ^18^, ^19^ However, it is still unclear if or how circulating B cells respond to such local respiratory virus infections. To address this, B cells were purified from peripheral blood mononuclear cells (PBMC) before and after experimental RV infection (day 3 and day 7) from a group of healthy human subjects without asthma or allergies from a previously reported study^29^ (Figure 1A, Figure S1). Characteristics of the healthy subjects are described in Table S1. The successful inoculation and development of infection of all participating subjects were confirmed previously by RV strain‐specific PCR in the nasal lavage and lower airways (see Figure S2 in the Online Appendix S1 section).^29^ RNA‐sequencing revealed early upregulation of genes enriched for pathways related to interferon‐induced antiviral responses and antibody responses on day 3 compared to baseline, while on day 7, significantly differently expressed genes mainly enriched for regulatory pathways (Figure 1B). Expression of most antiviral genes peaks on day 3 followed by downregulation by day 7 (Figure 1C). Furthermore, genes encoding pro‐inflammatory cytokines were upregulated on day 7 after infection, including *TNF* (TNF^30^), *IL*‐*6* (IL‐6^31^, ^32^) and *VEGFA* (VEGF‐A^33^) which have known functions in B‐T cell interactions or germinal centre development (Figure 1D). Additionally, genes involved in response to unfolded protein were also upregulated on day 7, probably reflecting increased protein expression (Figure 1E). Since access to samples from experimentally infected individuals was limited, antiviral response and inflammatory cytokine gene expression was also confirmed in B cells purified from *in vitro* infected PBMC (Figure 1F). In line with *in vivo* results, all genes were upregulated by RV stimulation compared to control. Interestingly, viral RNA level increased by day 3 in a virus‐dose dependent manner and was decreased again by day 7 (Figure 1G). To assess whether the observed increase comes from actual virus RNA production and does not represent continued uptake of viral particles, B cells were washed three times after 4 h of RV exposure to remove viral particles from the cell culture supernatant. The observed increase after 72 h suggests that transient infection of B cells takes place in this culture system (Figure 1H). Since cytoplasmic viral RNA might influence B‐cell responses *in vivo*, we used a highly sensitive PCR^34^ to measure RV‐derived RNA in B cells sorted from experimentally infected individuals. While we did not have all three time points available for each individual, RV RNA was detected in at least one sample from each individual with more than one time point (Figure 1I) and sequences aligned with EU126679.1 (HRV 16 5′ UTR) reference sequence (Figure S3). Interestingly, 42GH had a positive signal at baseline aligning with another strain (RV‐A29), likely representing an asymptomatic infection^3^, ^4^, ^5^ which does not result in upregulation of antiviral response genes.^28^ These results show for the first time the response of circulating B cells in RV‐infected healthy individuals and suggests that B cells interact with RV *in vivo* in humans during experimental RV infection.

### 3.2 Peripheral B cells are dependent on external type‐I‐IFN to reach an antiviral activation state

**FIGURE 2:**
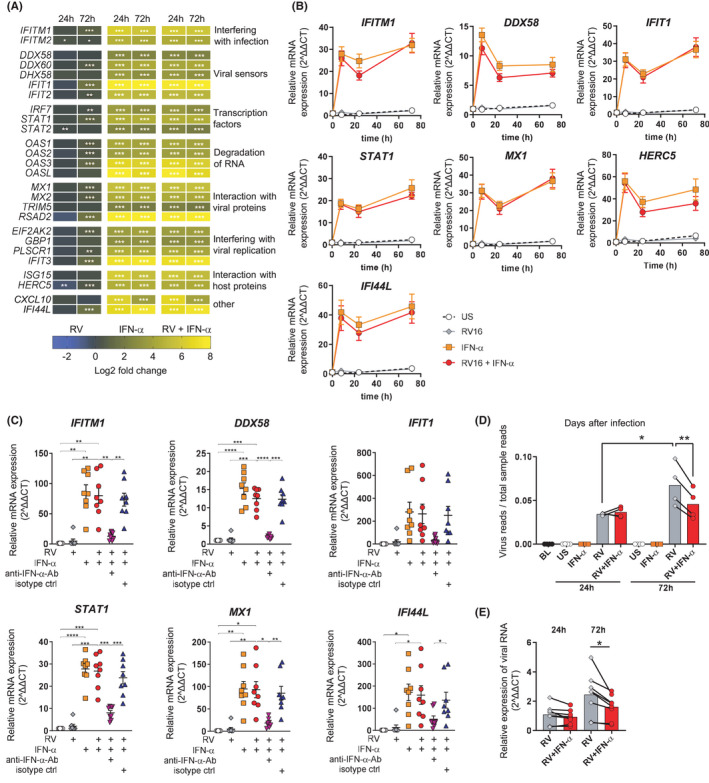
Peripheral B cells are dependent on external type‐I‐IFN to reach an antiviral activation state. Purified B cells from healthy individuals were stimulated with RV, IFN‐α and RV+IFN‐α; gene expression analysed using RNA‐sequencing (A, D, *n* = 4) or qPCR (B, C, E, *n* = 7). A, Gene expression change shown for interferon responsive genes (GO: 0051607 ‘Defense response to virus’). B, C, Gene expression of antiviral genes compared to baseline (B) or to US at 8 h (C). D, Virus reads in RNA‐sequenced samples normalized to total sample reads, paired *t* test, *P < .05, **P < .01. E, Viral RNA expression assessed using quantitative PCR. Individual colours indicate different stimulation conditions: US, RV, IFN‐α, RV+IFN‐α, RV+IFN‐α+IFN‐α‐blocking antibody, RV+IFN‐α+isotype control antibody, repeated measures one‐way ANOVA, *P < .05, **P < .01; ***P < .001; ****P < .0001, Values are means ± SEM, *n* = 8. RV was always used at MOI of 10

While B cells isolated from periphery of infected individuals or stimulated in total PBMCs showed a robust antiviral response, it is unknown whether B cells are dependent on interactions with other cell types or can also mount an antiviral response to RV by themselves. To address this in more detail, RNA‐sequencing was performed on pure B cells stimulated with either RV or IFN‐α alone, or in combination, and antiviral gene expression was assessed. In response to RV alone, B cells only showed low‐level expression of antiviral genes late after stimulation (72 h) (Figure 2A), setting them apart from other antigen‐presenting cells, such as monocytes,^35^, ^36^ dendritic cells^35^, ^37^ or macrophages.^38^, ^39^ Surprisingly, while type‐I IFN receptors were expressed, no IFN was expressed in B cells at either time points (see Figure S4), suggesting that the expression of antiviral genes was dependent on external IFN‐α stimulation (Figure 2A). Real‐time PCR revealed rapid upregulation and stable expression of *IFITM1*, *DDX58*, *STAT1*, *IFIT1*, *MX1*, *HERC5* and *IFI44L* (Figure 2B), genes whose protein products all interfere with central stages of the viral life cycle, such as detection and degradation of viral RNA.^40^, ^41^, ^42^, ^43^, ^44^, ^45^, ^46^, ^47^, ^48^, ^49^ Antibodies neutralizing IFN‐α inhibited expression of these antiviral genes (Figure 2C). In line with these results, IFN stimulation slightly decreased viral RNA levels in RV‐infected B‐cell cultures (Figure 2D,E). In conclusion, whereas B cells alone are unable to mount an antiviral response upon exposure to RV, a robust and rapid antiviral response was induced upon external stimulation with a type‐I‐IFN.

### 3.3 Rhinovirus stimulation drives plasmablast differentiation and induces a strong antiviral response in plasmablasts

**FIGURE 3:**
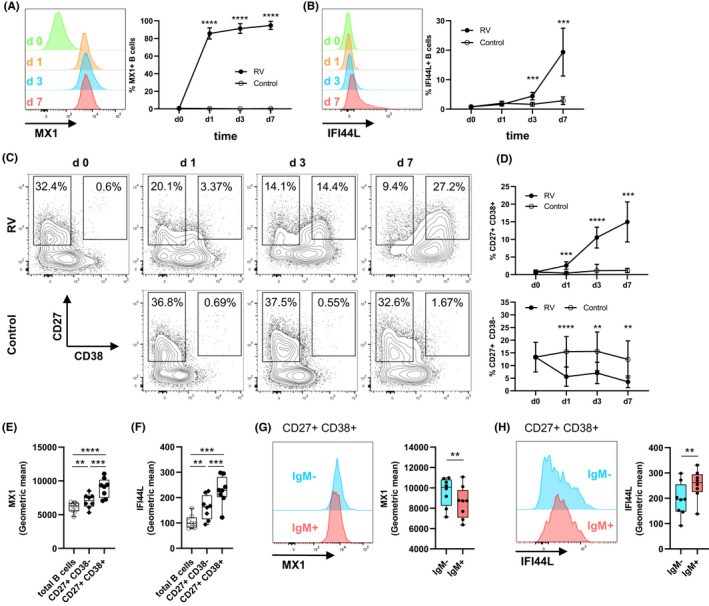
Rhinovirus stimulation drives plasmablast differentiation and induces strong antiviral response in plasmablasts. PBMCs from healthy human subjects were stimulated *in vitro* using RV. B‐cell markers, MX1 and IFI44L were analysed at baseline, day 1, day 3 and day 7 using flow cytometry. A, Expression of MX1 in total CD19+ B cells. B, Expression of IFI44L in total CD19+ B cells. C, Representative staining of CD27 and CD38, gated on total CD19+ B cells showing gates for CD27+ memory B cells and CD27+ CD38+ plasmablasts. D, Cumulative data showing percentage of CD27+ and CD27+ CD38+ B cells over time, *n* = 8. E‐H, Level of MX1 and IFI44L expression shown as geometric mean for total B cells, CD27+ CD38−, and CD27+ CD38+ cells (E, F) and for CD27+ CD38+ IgM+/IgM− plasmablasts (G, H)

Next, we aimed to confirm expression of antiviral response genes on protein level as well as timeline of expression in total B cells. As the upregulated antibody responses on day 3 after *in vivo* RV infection suggest B‐cell activation and differentiation (Figure 1B), we also asked whether expression of antiviral proteins differed between B‐cell subsets. Therefore, we combined staining of B‐cell surface markers with intracellular antibody staining for antiviral proteins using flow cytometry. Expression of two antiviral proteins was measured from which we expected different expression patterns: MX1, a protein rapidly induced by type‐I interferon which is interfering with viral replication,^44^ as well as IFI44L, recently shown to negatively regulate cellular responses after virus infections.^50^ MX1 was expressed in more than 80% of total B cells 24 h after RV stimulation of PBMCs (Figure 3A). In line with mRNA expression (Figure 1F), percentage of MX1+ B cell increased and stayed high for seven days after *in vitro* infection (Figure 3A). In contrast, IFI44L was only expressed starting at day 3, reaching approximately 20% of B cells by day 7 (Figure 3B). CD19+ B cells were then subgated for the expression of memory B‐cell marker CD27, as well as CD38, found on activated B‐cell subsets such as immature B cells and plasmablasts (Figure 3C). While number of total CD19+ B cells was unchanged over time (Figure S5A), percentage of CD27+ CD38+ plasmablasts from total CD19+ B cells increased from approximately 0.75% at baseline to more than 15% on day 7 after infection (Figure 3D). CD27+ memory B cells decreased compared to control (Figure 3D), suggesting partial differentiation into CD27+ CD38+ plasmablasts. Relative protein expression was compared among different B‐cell subsets, showing that plasmablasts have the highest expression MX1 (Figure 3E) and IFI44L (Figure 3F). MX1 and IFI44L were also differentially expressed in plasmablasts depending on the isotype, with switched IgM‐plasmablasts having higher MX1 (Figure 3G) and lower IFI44L levels (Figure 3H) compared to IgM+ plasmablasts. In summary, the antiviral response was most upregulated in the increasing subset of CD27+ CD38+ plasmablasts.

### 3.4 Upregulation of pro‐inflammatory cytokines in B cells is dependent on co‐stimulation with RV and IFN‐α

**FIGURE 4:**
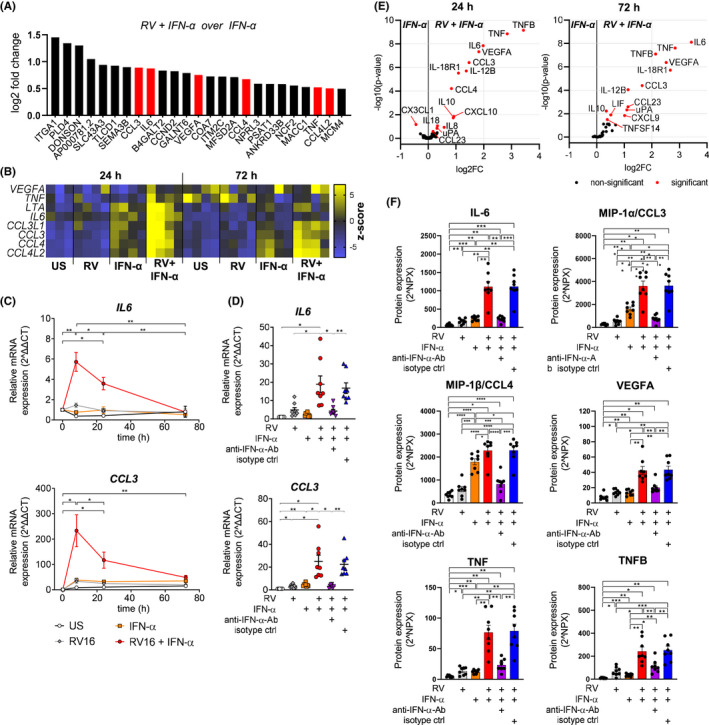
Upregulation of pro‐inflammatory cytokines in B cells is dependent on stimulation with both, RV and IFN‐α. A, 25 most differentially upregulated genes between *in vitro* stimulation with IFN‐α, and IFN‐α+RV, respectively (at 24 h), cytokine genes are marked red, *n* = 4. B, Gene expression of all significantly upregulated cytokine genes 24 and 72 h after stimulation, *n* = 4. C, D, Gene expression of antiviral genes compared to baseline (C) or to US at 8 h (D) measured using real‐time PCR, *n* = 8. E, Volcano plot shows differential protein expression of a total of 92 inflammatory cytokines between RV, and RV+IFN‐α on day 3 after stimulation, *n* = 8. F, Relative protein expression on day 3 after stimulation shown in linear scale (2^NPX), *n* = 8. Colours indicate different stimulation conditions: US, RV, IFN‐α, RV+IFN‐α, RV+IFN‐α+IFN‐α‐blocking antibody, RV+IFN‐α+isotype control antibody. Repeated measures one‐way ANOVA. All values are means ± SEM. RV: RV‐A16, IFN‐α2 (100 ng/ml) was used. *P < .05, **P < .01

While we described above that antiviral gene expression was found to be driven mainly by IFN‐α stimulation, it is currently unknown whether additional stimulation with RV leads to distinct B‐cell features which might play a role during active virus infections. To address this, we searched for highest upregulated genes in B cells stimulated with RV+IFN‐α compared to IFN‐α alone. Strikingly, eight genes encoding pro‐inflammatory cytokines were upregulated (Figure 4A,B). Expression of *IL*‐*6* and *CCL3* peaked 8 h after infection (Figure 4C). IFN‐α‐neutralizing antibodies abolished this effect, showing expression of these inflammatory cytokines is dependent on both stimulants, RV and IFN‐α (Figure 4D). Using a proximity extension assay including 92 inflammatory mediators, protein expression of MIP‐1α (*CCL3*), MIP‐1β (*CCL4*), IL‐6, TNF, TNFB (*LTA*) and VEGFA was confirmed (Figure 4E). With exception of MIP‐1α/β, all these cytokines were only induced after co‐stimulation with IFN‐α and RV (Figure 4F). Taken together, these data suggest that inflammatory cytokine genes detected *in vivo* and *in vitro* (Figure 1) were induced in response the co‐stimulatory effect of IFNs and direct RV exposure on B cells.

### 3.5 The peripheral B‐cell response to RV infection is elevated in asthma

**FIGURE 5:**
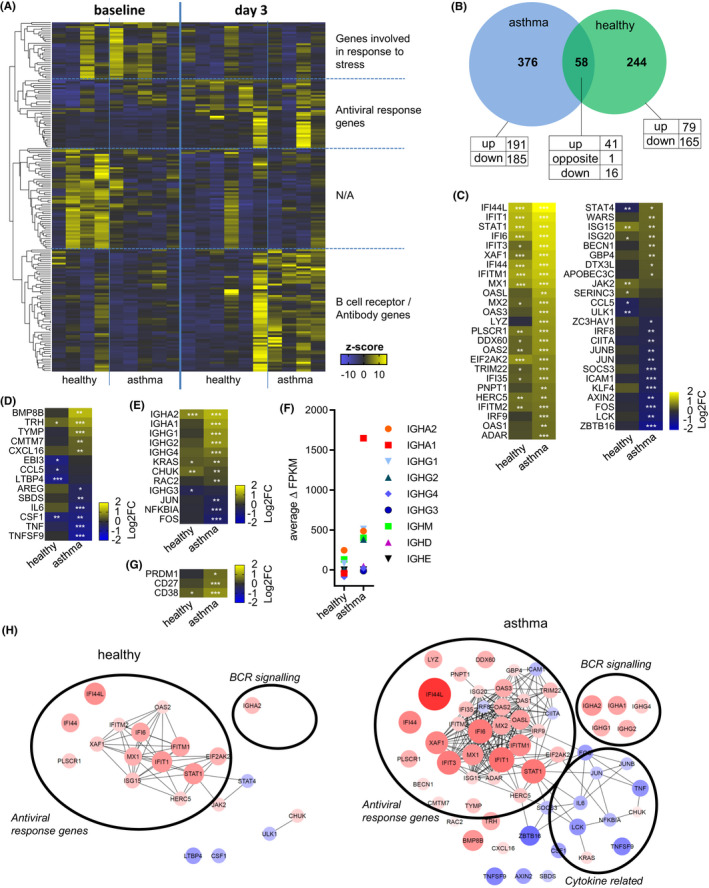
The peripheral B‐cell response to RV infection is elevated in asthma. A, Full RNA‐sequencing from purified B cells at baseline and 72 h after experimental infection. Top 100 genes most differentially expressed between healthy and asthma. Pathway enrichment shown for main clusters, *n* healthy =4 (baseline)/6 (day 3); *n* asthma =5 (baseline)/4 (day 3). B, Venn diagram shows differentially expressed genes on day 3 over baseline for asthma patients and healthy subjects. (C, D, E and G) Log2 fold change (‘log2FC’) of differentially expressed antiviral response genes (GO:0009615) (C), cytokine genes (D), BCR‐signalling genes (excluding individual V(D)J‐genes, E) and plasmablast‐related marker genes (G) in B cells isolated from healthy subjects and asthma patients. F, Average increase in heavy chain expression for individual heavy chain genes for healthy and asthma. H, Satellite plot showing known interactions of upregulated gene families

While RV infections are often asymptomatic or lead to mild symptoms in individuals with no underlying disease,^3^, ^4^, ^5^ they present the most common cause for exacerbations of asthma.^25^ Furthermore, skewed and less efficient antiviral immune responses were observed in patients with asthma.^26^, ^27^, ^29^, ^36^, ^38^, ^51^, ^52^ To address whether the reported B‐cell response to acute RV infection is dysregulated in individuals with asthma, RNA‐sequencing was also performed in B cells sorted from asthma patients before and on day 3 after experimental RV infection and compared to healthy individuals’ gene expression changes (Figure 5A). While genes directly involved in cellular antiviral response were upregulated in both, healthy subjects and patients with asthma, antibody‐related genes were predominantly upregulated in asthma (Figure 5A). A total of 58 genes were changed in both subject groups (Figure 5B), most of them involved in various aspects of antiviral response, including viral sensors (*IFIT1*, *DDX60*), transcription factors (*STAT1*, *IRF9*), degradation of viral RNA (*OAS2*, *OAS3*, *OASL*), interacting with viral proteins (*MX1*, *MX2*), or regulation of antiviral response (*ISG15*, *IFI44L*)^48^, ^53^ (Figure 5C). Expression of these genes in B cells was similar after *in vitro* stimulation with IFN‐α (Figure S6), and they are also upregulated after experimental RV infection in whole blood.^28^ The number of antiviral genes that were expressed in B cells from asthma patients was higher, and these were more upregulated compared to B cells from healthy subjects. In addition, expression of pro‐inflammatory cytokines including *IL*‐*6* and *TNF* was slightly downregulated upon infection (Figure 5D) while BCR‐signalling genes were significantly upregulated (Figure 5E). Moreover, the average increase in heavy chain expression (Figure 5F) and expression of plasmablasts markers were more upregulated in patients with asthma (Figure 5G). Interestingly, expression of type‐I IFN receptors was similar between healthy individuals and those with asthma (data not shown). Interaction network analysis using STRING^54^ revealed a significantly larger and extensive network of differentially regulated genes with known interactions in the asthma group in response to RV infection (Figure 5H). In summary, total number of antiviral and antibody‐related genes in asthma patients were higher and these were more upregulated compared to infected non‐asthmatics individuals suggesting B‐cell response is broad and exaggerated at the gene expression level in asthma.

## 4 DISCUSSION

We report the first comprehensive analysis of the gene regulation in B cell following RV infection *in vivo* in human. This B‐cell response was mainly characterized by an IFN‐induced antiviral gene programme, transient production of viral RNA in B cells, expression of antiviral proteins and pro‐inflammatory cytokines, and was dysregulated in asthma patients.

Here, we studied B cells sorted from the peripheral blood of healthy human subjects that were experimentally intranasally infected with RV‐A16. We chose RNA‐sequencing of highly purified B cells as an unbiased approach to study the suspected antiviral response in circulating B cells. On day 3, circulating B cells showed upregulated antibody and interferon signalling‐induced antiviral genes. While this IFN‐driven antiviral response was mostly downregulated on day 7 after infection, a pro‐inflammatory cytokine response was upregulated instead. These results are comparable to a study showing early IFN‐driven antiviral responses in whole blood, followed by secondary inflammatory responses during influenza infection in humans.^55^ We found a relatively high antiviral gene expression variability between individual subjects in which seems to replicate results of an earlier study where gene expression was analysed after experimental infection with RV‐A39 in full blood.^28^ While a prior study showed a positive correlation of viral load in nasal lavage with neutralizing antibody titre after experimental RV infection,^29^ our subject number was too low to determine whether there is a direct relationship between viral load in different tissues and gene expression in peripheral B cells. As we worked with residual samples from a previous study^29^ and hence the number of *in vivo* samples was limited, we confirmed expression of selected antiviral and pro‐inflammatory cytokine genes *in vitro* with similar results. In addition, the upregulated antiviral genes that we detected in purified B cells also overlap to a large extent with genes upregulated in whole blood after RV infection.^28^, ^56^ The stimulation driving this gene expression programme seems not to originate from cells of the blood, as IFN expression cannot be detected in whole blood after experimental RV infection.^28^ Interestingly, RV is sometimes detected in tonsils^17^ where B cells make up about 60% of total cells.^16^ Furthermore, B cells were also found in nasal tissue of individuals infected with RV while absent in non‐infected controls.^15^ Therefore, it seems likely that circulating B cells get stimulated by IFNs released either by phagocytes in airway‐associated lymphoid tissue or secreted by airway epithelial cells when they traffic through airway tissue close to infection sites.^14^

We showed that circulating B cells isolated from experimentally infected subjects carried RV RNA, suggesting that direct interactions of B cells with RV virions occur in infected humans. In addition to IFN stimulation, this interaction with RV seemed crucial for B cells to express the pro‐inflammatory cytokines reported in this study. Detection of viral RNA on B cells might also suggest that B cells acted as antigen‐presenting cells during RV infection, as shown during viral infections in mouse models.^21^, ^22^ *In vivo* interaction of B cells with other viruses, including influenza,^57^ respiratory syncytial virus^58^ and dengue virus,^59^ was also described. In patients having severe dengue virus infection, single‐cell sequencing revealed that more than 40% of B cells carried viral RNA.^59^ However, it is not possible to assess from our data whether B cells isolated from experimentally infected individuals simply transported viral particles bound to surface receptors as was reported before for virus‐like‐particles,^21^ or whether they internalized virions and were infected. Nonetheless, our *in vitro* data suggest that at least transient viral RNA production can occur in B cells.

Surprisingly, unlike other antigen‐presenting cells,^35^, ^36^, ^37^, ^38^ B cells did not express IFNs in response to RV and therefore seem to rely on signals from other by standing cells to bring them into an antiviral state. This lack of response seems not limited to RV, as also B cells exposed to respiratory syncytial virus,^58^ as well as circulating B cells isolated from patients suffering from severe dengue virus infections were not expressing interferon genes.^59^ In contrast, B cells chronically infected with EBV expressed *IFNA2* (IFN‐α) implying there is no general deficiency in B cells to secrete IFNs.^60^ Since many genes involved in viral recognition, including viral ssRNA‐sensing TLR7, and downstream IFN‐α‐inducing transcription factor IRF7 were expressed in our experiments, negative regulation to prevent IFN secretion could take place at translational or post‐translational levels.^61^

Addressing the antiviral response on a protein level revealed a different picture: while the antiviral protein MX1^44^ was rapidly expressed by B cells upon infection, IFI44L, a negative regulator of the antiviral response,^50^ peaked on day 7 when transient infection already decreased again. Upon RV stimulation *in vitro* B cells rapidly differentiated into CD27+ CD38+ plasmablasts, with MX1 and IFI44L also upregulated highest in this subset. These two processes are probably both driven by the same stimulation, as the type‐I IFN signalling‐induced expression of these antiviral proteins also plays an important role during B‐cell differentiation,^9^, ^10^, ^11^ antibody class‐switching^12^ and antibody secretion.^9^, ^13^ Similarly, an elevated expression of antiviral genes was also accompanied by increased expression of antibody genes and plasmablast markers *CD27*, *CD38* and *PRDM1* in asthma patients on day 3 after infection. Therefore, the exaggerated but less efficient antibody response found in asthma^26^, ^27^ might partially be explained by B cells stimulated with elevated type‐I IFN levels in these patients.

It has often been reported that some patients with asthma show a delay or even deficiency in virus induction of antiviral IFNs when their cells are studied *ex vivo* (reviewed in^52^). The observed elevated antiviral and antibody gene expression in asthmatic subjects in this study suggests that these B cells may be stimulated by higher IFN levels. Indeed, greater IFN levels during *in vivo* infection have been reported in a similar study^62^ in which higher viral loads in subjects with asthma were also observed.^63^ In addition, a recent study on naturally RV‐infected children with asthma also supports the theory that deficient early IFN responses may lead to unchecked virus replication, leading to greater virus loads, which subsequently results in exaggerated IFN responses.^64^ Greater virus loads were also observed in the subjects with asthma providing PBMC for the present study (see Figure S2), however, with the much smaller subject numbers studied these differences were not statistically different. Overall, our results suggest that higher viral loads in patients with asthma could lead to an exaggerated antiviral response in the periphery.

A limitation of our study is that gene expression was only measured 3 days after experimental infection, while type‐I‐IFNs are already secreted within hours. Sampling at earlier, as well as later time points after remission, might help to better understand in which step the antiviral response is dysregulated in subjects with asthma. However, it is practically difficult to obtain these materials in a human *in vivo* study. In addition, given the relatively low number of subjects, the significant genes should be carefully reviewed and verified in larger cohorts. Nonetheless, the genes that were differentially expressed in B cells after RV infection of healthy individuals in the current study are similar to those found upregulated in earlier studies.^28^, ^65^

The peripheral B‐cell responses reported here were mostly dependent on type‐I‐IFN stimulation, as well as exposure to RV‐A, which is a positive‐sense RNA virus. Therefore, it can be expected that our findings are not restricted to infections with RV‐A species only and might represent a general response towards different RV species and comparable viruses infecting the respiratory tract. Given the frequent incidence of such infections and because of the current SARS‐CoV‐2 pandemic, antibody responses to respiratory viruses are studied intensively. It could be important to also focus on B‐cell functions other than antibody production, including cytokine production and antigen presentation to fully understand the role of B cells responding to these pathogens.

## CONFLICT OF INTEREST

Dr. Willem van de Veen reports grants from Novartis Forschungsstiftung, and grants from Promedica Stiftung, outside the submitted work. Dr. Sokolowska reports grants from Swiss National Science Foundation, grants from GSK, and grants from Novartis, outside the submitted work. Dr. Glanville has a patent US9937252B2 'Induction of cross‐reactive cellular response against rhinovirus antigens' pending. Dr. Gern reports grants from NIH, during the conduct of the study; personal fees from Regeneron, personal fees and stock options from Meissa Vaccines Inc, and personal fees from MedImmune/AstraZeneca, outside the submitted work. In addition, Dr. Gern has patents for Methods of Propagating Rhinovirus C in Previously Unsusceptible Cell Lines, and for Adapted Rhinovirus C. Dr. Papadopoulos reports personal fees from Novartis, Nutricia, HAL, MENARINI/FAES FARMA, SANOFI, MYLAN/MEDA, BIOMAY, AstraZeneca, GSK, MSD, ASIT BIOTECH, Boehringer Ingelheim, grants from Gerolymatos International SA, and grants from Capricare, outside the submitted work. Dr. Akdis reports grants from Allergopharma, Idorsia, Swiss National Science Foundation, Christine Kühne‐Center for Allergy Research and Education, European Commission’s Horizon's 2020 Framework Programme, Cure, Novartis Research Institutes, Basel, AstraZeneca, Switzerland, Scibase, Stockholm, advisory role for Sanofi/Regeneron, Glaxo Smith‐Kline, Novartis. Dr. Johnston reports personal fees from Virtus Respiratory Research, personal fees from Myelo Therapeutics GmbH, personal fees from Bayer, personal fees from Novartis, personal fees from Boehringer Ingelheim, personal fees from Gerson Lehrman Group, personal fees from resTORbio, personal fees from Bioforce, personal fees from Lallemand Pharma, outside the submitted work; in addition, Dr. Johnston has a patent Wark PA, Johnston SL, Holgate ST, Davies DE “Anti‐virus therapy for respiratory diseases”, UK patent application No. GB 0405634.7, 12 March 2004, with royalties paid, a patent Wark PA, Johnston SL, Holgate ST, Davies DE “Interferon‐Beta for Anti‐Virus Therapy for Respiratory Diseases”, International Patent Application No. PCT/GB05/50031, 12 March 2004, with royalties paid, and a patent Davies DE, Wark PA, Holgate ST, Johnston SL “Interferon Lambda Therapy for the Treatment of Respiratory disease”, UK Patent application No. 6779645.9, granted 15th August 2012, licensed. Dr. Nadeau reports grants from National Institute of Allergy and Infectious Diseases (NIAID), National Heart, Lung, and Blood Institute (NHLBI), National Institute of Environmental Health Sciences (NIEHS), and Food Allergy Research & Education (FARE); Director of World Allergy Organization (WAO), Advisor at Cour Pharma, co‐founder of Before Brands, Alladapt, Latitude, and IgGenix; and National Scientific Committee member at Immune Tolerance Network (ITN), and National Institutes of Health (NIH) clinical research centers, outside the submitted work; in addition, Dr. Nadeau has the following patents: "Special Oral Formula for Decreasing Food Allergy Risk and Treatment for Food Allergy," (with royalties paid to Before Brands and Alladapt), "Granulocyte‐based methods for detecting and monitoring immune system disorders," (issued), "Methods and Assays for Detecting and Quantifying Pure Subpopulations of White Blood Cells in Immune System Disorders," (issued), "Microfluidic Device and Diagnostic Methods for Allergy Testing Based on Detection of Basophil Activation," (pending). The other authors declare that they have no potential conflict of interest.

## AUTHOR CONTRIBUTIONS

OFW had primary responsibility for framework of the study, data analysis and manuscript preparation. OFW, WV, MS, KJ, BS, SLJ, JEG, NGP, CA, KN and MA contributed to the conception and design of the study and interpretation of results, and MA supervised the project. OFW, KJ, PS and DM performed the experiments. SLJ, SDM, TK, NG and PM performed the experimental infection study. OFW and GT conducted data analysis and interpretation of the results. All authors contributed to the revision of the manuscript.

## Supporting information

Supplementary Material
