# Supplementary material for: Experimental rhinovirus infection induces an antiviral response in circulating B cells which is dysregulated in patients with asthma
Source: Allergy. 2021 Jul 16;77(1):130–42. doi: 10.1111/all.14985 (PMC10138744; doi:10.1111/all.14985)
Supplement: Supplementary file 1 — Supplementary Material [file ALL-77-130-s001.docx]

**Supporting information**

**Study Design**

RV-A16 experimental infection was induced in RV-A16 seronegative atopic asthmatic (n_baseline_=5, n_day3_=4) and healthy non-atopic non-asthmatic (n_baseline_=4, n_day3_=6, n_day7_=6) subjects (see **Table S1** for subject characteristics)^1^. The difference in the number of subjects before and after infection was related to the availability of the samples from the previously performed studies^1^. Clinical and atopic status was assessed by medical anamnesis, skin prick testing, measurements of serum IgE and lung function testing including peak expiratory flow (PEF), forced expiratory volume in 1 s (FEV_1_), forced vital capacity (FVC), and histamine challenge. Asthmatics were only taking short-acting β_2_ agonists if needed, while healthy subjects did not take medication. Subjects were free of common cold symptoms for at least 6 weeks before the start of the study. There were no severe adverse events upon infection and asthmatic patients did not need to take inhaled or oral steroids during the study. The successful inoculation and development of infection of all participating subjects was confirmed previously by RV strain-specific PCR in the nasal lavage and lower airways (**Figure S2**)^1^. All individuals were non-smokers.

Peripheral blood mononuclear cells (PBMC) were isolated and cryopreserved before and after 3 and 7 days of acute infection. Samples were taken ~14 days before infection for baseline timepoint, and diaries were kept to record symptoms and home lung function throughout the study. All subjects gave written informed consent. The study was approved by the St Mary's National Health Service Trust Research Ethics Committee.

**Experimental Infection with RV-A16**

Infection was induced by using 10,000 tissue culture 50% infective dose RV-A16 on day 0 by nasal spray as described^2^. After inoculation, subjects returned home. Infection was confirmed for all subjects by rhinovirus specific Taqman RV RT-PCR in nasal lavage (see **Figure S2**). In addition, virus levels were assessed in induced sputum and BAL. Confirmation of infection among the here presented subjects was performed as part of a previous study^1^.

**Skin Prick Testing and Serum IgE**

Atopy was determined by skin prick testing to common aeroallergens: six grass pollen mix; house dust mite; cat; dog; *Aspergillus fumigatus*; *Cladosporium herbarum*; *Alternaria alternata*; and birch, three tree and nettle pollen (ALK Abello)^1^. One wheal 3 mm greater than negative control or total IgE > 110 units/ml was considered diagnostic of atopy.

**Sample preparation**

PBMC from experimentally infected individuals were thawed and washed with complete RPMI 1640 medium supplemented with L-glutamine (2 mmol/l), MEM vitamin, penicillin (100 U/ml), streptomycin (100 lg/ml), kanamycin, nonessential amino acids, sodium pyruvate (Life Technologies, Carlsbad, CA, USA), and 10% heat-inactivated fetal calf serum (FCS; Invitrogen, Carlsbad, CA, USA). Then, PBMC were rested in cRPMI for 30 min at 37°C on a roller at low speed. Afterwards, PBMC were centrifuged and resuspended in staining buffer (PBS, 0.5% BSA). PBMC were stained with anti-CD19 PE/Cy7-antibody (Biolegend, clone HIB19), anti-CD3 BV510-antibody (Biolegend, clone UCHT1) and viability dye eFluor780 (Thermo Fisher). Viability dye was 1:1000 diluted in staining buffer. Cells were incubated for 15 min at 4°C, washed and filtered prior to sorting. CD19+ B cells were sorted using a BD ARIA IIIu (Becton Dickinson, Franklin Lanes, NJ) (see **Figure S1A** and **B),** and B cell purity was measured (purity: 98.8% +/- 0.86 SD for baseline samples, 98.7% +/- 0.5 SD day 3 samples, 98.9% +/- 0.8 SD day 7 samples). Sorted cells were lysed immediately using RLT plus buffer (QIAGEN, Hilden, Germany). Cell lysates were stored at -80°C until total RNA was isolated using RNeasy Plus Micro Kit (QIAGEN). RNA was stored at -80°C until it was analysed.

**Flow cytometry staining**

PBMCs were cultured for up to 7 days at 37° C in humidified 5% CO_2_ with or without viable virus (MOI10). Cells were stained first with Zombie Yellow™ Fixable Viability Kit (Biolegend), followed by surface marker staining with anti-CD19 PE/Cy7-antibody (Biolegend), Mouse anti-human CD38 antibody BV785 (Biolegend), Mouse anti-human CD27 antibody BV510 (BD Biosciences), and Mouse anti-human IgM antibody PerCP-Cy5.5 (Biolegend). Intracellular staining was performed with DyLight405-conjugated Mouse anti-human IFI44L antibody (Antibodies-online Inc.) and Mouse anti-human MxA antibody AF647 (Abcam) was performed using Fixation/Permeabilization Solution Kit (BD Biosciences) according to the manufacturer’s protocol.

**Cell culture methods**

PBMCs were isolated by Ficoll (Biochrom, Berlin, Germany) density gradient centrifugation from peripheral venous blood. B cell isolation was performed by labeling non-B cells using a cocktail of biotin-conjugated antibodies against CD2, CD14, CD16, CD36, CD43 and CD235a and anti-biotin microbeads (Miltenyi Biotec, Bergisch Gladbach, Germany) followed by immunomagnetic separation (AutoMacs; Miltenyi Biotec). The purity of isolated CD19+ B cells was routinely >97%. Stimulation of untouched B cells was performed at a cell density of 1 Mio/ml with multiplicity of infection (MOI) of 10 infectious units (IU) per cell (MOI10) of RV-A16 (Virapur, San Diego, CA, USA) and/or 100µg/ml IFN-α2 (Peprotec, Cat. Nr. 300-02AA, product temporarily discontinued) or UV irradiated RV-A16 (15 min from 2 cm distance, 254nm). In this study, IFN-α2 was used as a representative for type-I-IFNs and is mainly produced by hematopoietic cells ^3^, including plasmacytoid dendritic cells ^4^ often found in lung draining lymph nodes. Cells and supernatants were harvested, and RNA was isolated from cell pellets as described above.

**Real-time PCR**

cDNA was synthesized with RevertAid RT Reverse Transcription KIT (Thermo Fisher Scientific) according to manufacturer’s protocol. cDNAs were amplified with Maxima SYBR Green/ROX qPCR Master Mix (2X) (Thermo Fisher Scientific) and 0.6 µM of primers. Thermal cycling was performed with a two-step cycling protocol according to the manufacturer’s recommendations using a Quant Studio 7 Flex (Thermo Fisher Scientific). Expression was normalized with the housekeeping gene EF1α and relative quantification was performed using the comparative ΔΔCT method. RV copy number was determined using Human Rhinovirus Subtype 16 kit (PCRmax).

**PCR for RV detection**

High sensitivity detection of RV was performed using a previously described protocol^E5^. In short, RNA was isolated from peripheral B cells before or after experimental RV infection, transcribed to cDNA and used for the viral detection PCR. A two-step PCR protocol was performed with an experimentally determined detection limit of around ten viral copies in a biological sample^E5^. As a positive control, RV-B14 was used which is genetically very distinct from RV-A16^E6^ to be able to easily detect possible contaminations. Resulting bands at around 400 bp were excised and purified PCR products from gels were sequenced and aligned to sequences from the databank as described^5^.

**Quantification of cytokines**

Cytokines were quantitatively determined in cell-culture supernatants using OLINK targeted proteomics technology (Uppsala, Sweden). 92 inflammatory mediators were measured from B cell culture supernatants using proximity extension assay (inflammation panel, according to manufacturer’s protocol.

**Library preparation and RNA sequencing**

The quantity and quality of the isolated RNA was determined with a Qubit® (1.0) Fluorometer (Life Technologies, California, USA) and a Bioanalyzer 2100 (Agilent, Waldbronn, Germany) and samples with RNA-integrity number >7.0 were selected for next generation RNA sequencing (NGS). Library preparation for NGS was performed using the TruSeq Stranded mRNA Sample Prep Kit (Illumina, Inc, California, USA). Total RNA (100 ng) was poly-A enriched and reverse-transcribed into double-stranded cDNA. cDNA samples were fragmented, end-repaired and poly-adenylated before ligation of TruSeq adapters for demultiplexing. Fragments containing TruSeq adapters on both ends were selectively enriched with PCR. The quality and quantity of the enriched libraries was validated using Qubit® (1.0) Fluorometer and the Caliper GX LabChip(R) GX (Caliper Life Sciences, Inc., USA). The libraries were normalized to 10nM in Tris-Cl 10 mM, pH8.5 with 0.1% Tween 20.

The TruSeq SR Cluster Kit v4-cBot-HS or TruSeq PE Cluster Kit v4-cBot-HS (Illumina, Inc, California, USA) was used for cluster generation using 10 pM of pooled normalized libraries on the cBOT. Sequencing were performed on the Illumina HiSeq 4000 single end 125 bp using the TruSeq SBS Kit v4-HS (Illumina, Inc, California, USA). The sequencing data shown in this publication is archived in NCBI’s Gene Expression Omnibus with accession number GSE118875.

Overrepresentation analysis of Gene Ontology (GO) categories was performed using Bioconductor package goseq (v1.28.0) ^E7^ with the Wallenius approximation. Protein interaction network was prepared using STRING (v10) ^E8^ and further processed with Cytoscape (v3.2.1) ^E9^. Networks and pathway enrichment were generated using MetaCore version 6.3 (Thomson Reuters).

**Data analysis**

NGS was performed for the quantitative assessment of gene expression. Adapters and low-quality tails were trimmed from reads prior to read alignment. STAR aligner (v2.5.3a) ^E10^ was used to align the RNA-seq dataset to Ensembl genome build GRCh38.p10 (Release 89). Gene expression counts were calculated with feature counts from Bioconductor package Rsubread (v1.26.0) ^E11^. A gene was considered as expressed if, in at least one group, it had more than 10 counts in more than half of the replicates. Differential expressed genes were detected using generalized linear model with quasi-likelihood test implemented in Bioconductior package edgeR (v3.18.1) ^E12^. Genes with p-value <= 0.01 and log2 fold change >= 0.5 were used in this studyGene lists were created as described in the text or based on gene lists from NIAID ImmPort Resources (<http://www.immport.org/immport-open/public/reference/genelists>).

**Supplemental Information References:**

1. Message SD, Laza-Stanca V, Mallia P, Parker HL, Zhu J, Kebadze T, et al. Rhinovirus-induced lower respiratory illness is increased in asthma and related to virus load and Th1/2 cytokine and IL-10 production. Proc Natl Acad Sci U S A 2008; 105:13562-7.

2. Bardin PG, Sanderson G, Robinson BS, Holgate ST, Tyrrell DA. Experimental rhinovirus infection in volunteers. Eur Respir J 1996; 9:2250-5.

3. Ivashkiv LB, Donlin LT. Regulation of type I interferon responses. Nat Rev Immunol 2014; 14:36-49.

4. Xi Y, Finlayson A, White OJ, Carroll ML, Upham JW. Rhinovirus stimulated IFN-alpha production: how important are plasmacytoid DCs, monocytes and endosomal pH? Clin Transl Immunology 2015; 4:e46.

5. Wai-Ming Lee KG, Rose Vrtis, Tressa Pappas, Fue Vang, Iris Lee, and James E. Gern. Molecular Identification and Quantification of Human Rhinoviruses in Respiratory Samples. Rhinoviruses: Methods and Protocols, Methods in Molecular Biology 2015; 1221:25.

6. Palmenberg AC, Spiro D, Kuzmickas R, Wang S, Djikeng A, Rathe JA, et al. Sequencing and analyses of all known human rhinovirus genomes reveal structure and evolution. Science 2009; 324:55-9.

7. Young MD, Wakefield MJ, Smyth GK, Oshlack A. Gene ontology analysis for RNA-seq: accounting for selection bias. Genome Biol 2010; 11:R14.

8. Szklarczyk D, Morris JH, Cook H, Kuhn M, Wyder S, Simonovic M, et al. The STRING database in 2017: quality-controlled protein-protein association networks, made broadly accessible. Nucleic Acids Res 2017; 45:D362-d8.

9. Shannon P, Markiel A, Ozier O, Baliga NS, Wang JT, Ramage D, et al. Cytoscape: a software environment for integrated models of biomolecular interaction networks. Genome Res 2003; 13:2498-504.

10. Dobin A, Davis CA, Schlesinger F, Drenkow J, Zaleski C, Jha S, et al. STAR: ultrafast universal RNA-seq aligner. Bioinformatics 2013; 29:15-21.

11. Liao Y, Smyth GK, Shi W. The Subread aligner: fast, accurate and scalable read mapping by seed-and-vote. Nucleic Acids Res 2013; 41:e108.

12. Robinson MD, McCarthy DJ, Smyth GK. edgeR: a Bioconductor package for differential expression analysis of digital gene expression data. Bioinformatics 2010; 26:139-40.

**Supplemental Figures**


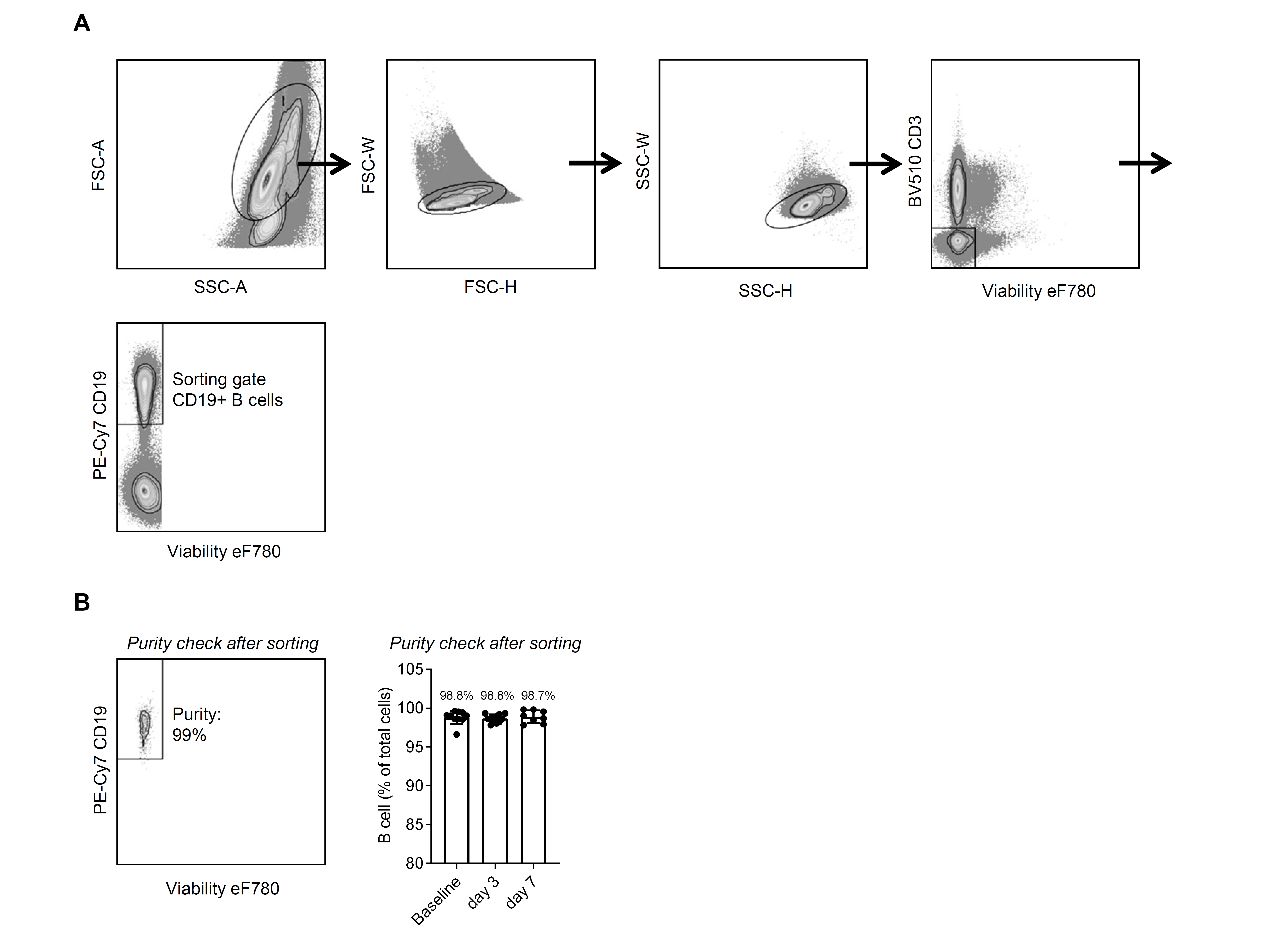


**Figure S1: Gating strategy for sorting and purity check of sequenced samples.** (A) Gating strategy for CD19+ B cells from PBMC samples. (B) Representative purity check staining, gated on total cells of sorted sample (left panel) and cumulative data for purity on baseline, day 3, and day 7 (right panel) are shown. Flow cytometry plots in (A) and (B) are representative data coming from one healthy donor (no 39).


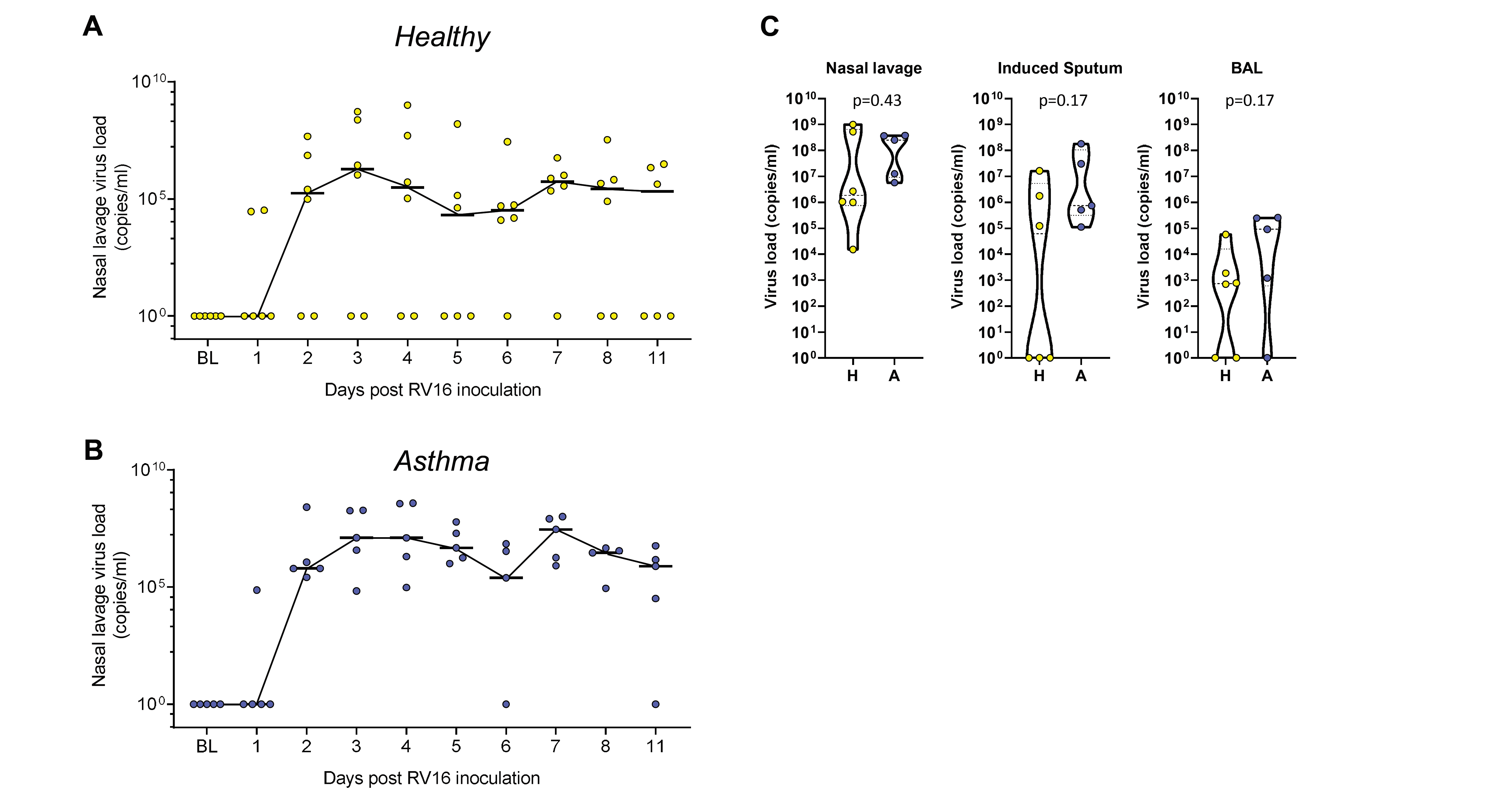


**Figure S2: Rhinovirus virus load in the upper and lower respiratory tract after experimental RV-A16 infection.** (A and B) Rhinovirus virus load (Log10 copies/ml) was determined by real-time PCR during experimental RV-A16 infection of healthy (●) and asthmatic (●) subjects in upper (nasal lavage, NL) and lower (induced sputum, IS and bronchoalveolar lavage, BAL) respiratory tract samples. NL was obtained on days 1–8 and 11 after inoculation with RV-A16 on day 0. IS was obtained on days 3 and 7 and BAL was obtained on day 4. The time course of virus loads in NL were similar in the healthy (●) and asthmatic (●) groups. Individual data points are shown, the solid bar represents the median virus load on each day. Virus load was undetectable in most subjects on day 1 but increased rapidly from day 2 to peak around day 2–4, consistent with viral replication in the respiratory tract. (C) Virus load for each donor at individual peak of infection. Median virus load was between 1–2 Logs greater in the asthmatic group in all three airway samples, but differences were not statistically significant (Mann-Whitney test).


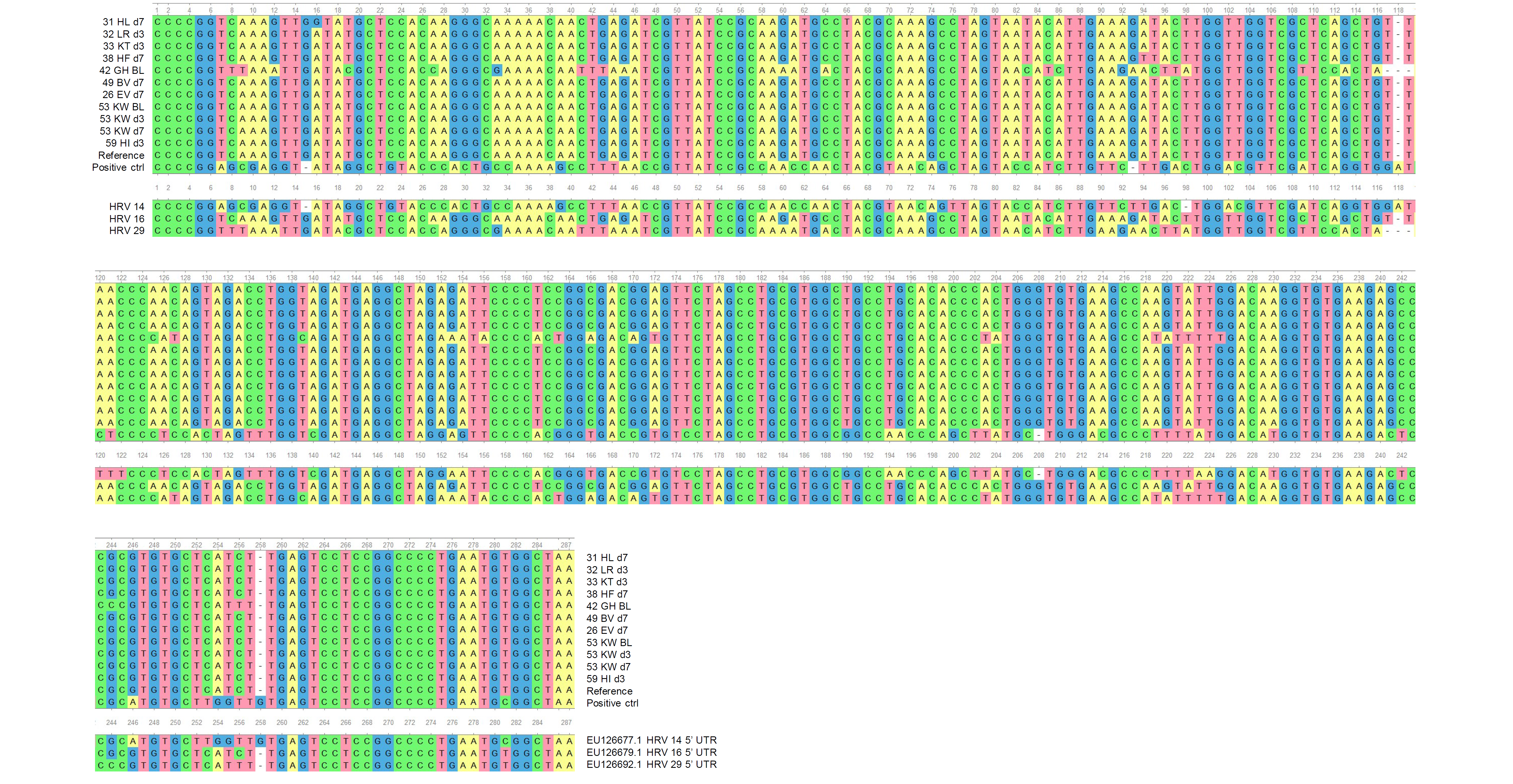


**Figure S3: Rhinovirus sequences from B cells of experimentally infected subjects**

Sequencing results from excised bands from Figure 6 (A) and (B) are shown. “Positive control” is RV-B14. “Reference” depicts an RV-A16 sample from the same lab that performed the experimental infection experiment and was processed at another timepoint and sequenced separately.

**Figure S4: Interferon genes are not expressed in B cells**

RNA-sequencing of purified B cells was performed after *in vitro*-stimulation with medium (US, unstimulated), RV-A16 and/or IFN-α2. Heatmap shows normalized gene expression (FPKM) of interferon-receptor genes and shows that interferon genes are not expressed at baseline (BL), 24 and 72h after stimulation in any condition.

**Figure S5: B cell number after in vitro stimulation of PBMC with RV-A16**

(A) Percentage of CD19+ B cells from total viable single cells after *in vitro* culture with medium control (US), or RV-A16 (RV) shows similar numbers in total B cell between the two culture conditions. (B) Percentage of IgM+ cells from total CD27+ CD38+ plasmablasts.

**Figure S6: Gene expression in B cells of experimentally infected individuals resembles expression of interferon-induced antiviral genes in cultured B cells.**

Gene expression change (“log2 FC”) from all differentially expressed genes after experimental RV infection of healthy (A) and asthmatic individuals (B) correlated to gene expression after *in vitro* stimulation with IFN-α. Genes of the Gene ontology pathway “Response to virus” (GO: 0009615) are labelled and marked orange.

**Table S1: Baseline characteristics of recruited asthmatic and healthy subjects**

| **Subject no.** | **Age** | **Sex** | **IgE units/ml** | **Skin prick tests**  **positive** | **Baseline FEV1 % predicted** | **Baseline histamine**  **PC20, mg/ml *** | **Baseline histamine**  **PC10, mg/ml** | **Allergy syndromes** |
| --- | --- | --- | --- | --- | --- | --- | --- | --- |
| ***Healthy (non-asthmatic, non-atopic)*** | | | | | | | | |
| 19 | 31 | F | 7 | Nil | 122 | 8.3 | 5.46 | Nil |
| 31 | 19 | M | 19 | Nil | 99 | 10.2 | 3.11 | Nil |
| 32 | 24 | F | 22 | Nil | 99 | > 32 (17.1) | 5.78 | Nil |
| 33 | 28 | F | 14 | Nil | 136 | > 16 (7.8) | 20 | Nil |
| 38 | 18 | M | 6 | Nil | 109 | 12.4 | 10.9 | Nil |
| 42 | 23 | F | 11 | Nil | 85 | > 16 (10.3) | 15.26 | Nil |
| 49 | 18 | F | 16 | Nil | 86 | > 16 (12.9) | 11.3 | Nil |
| Median | 23 | 2M / 5F | 13.6 |  | 106 |  | 10.3 |  |
| ***Atopic asthmatics*** | | | | | | | | |
| 26 | 24 | F | 229 | d(grade 3^C^)latex(6) | 83 | 0.228 | 0.139 | A, E |
| 39 | 19 | M | 162 | Nil | 105 | 2.83 | 0.794 | A |
| 53 | 22 | F | 212 | h(5) | 118 | 7.4 | 4.37 | A |
| 59 | 21 | F | 374 | g(4)h(4)a(3)b(3)  3t(3)c(6)d(4) | 104 | 1.37 | 0.886 | A, R |
| 62 | 22 | F | 355 | h(5)c(2) | 136 | 3.93 | 2.16 | A |
| Median | 22 | 1M / 4F | 266 |  | 109 | 3.16 | 1.66 |  |

g, grass pollen; h, house mite; cl, cladosporium; a, aspergillus; b, birch; 3t, 3 trees; n, nettle; c, cat; d, dog; alt, alternaria; A, asthma; R, rhinitis; E, eczema.

*One healthy subject had baseline histamine PC20 > 32 mg/ml (the maximum concentration used in this study) and three were unable to tolerate a dose of histamine greater than 16 mg/ml. Where this was the case the percentage fall in FEV1 is shown in parentheses alongside the maximum histamine concentration used.
